# Bayesian Profile Regression to Deal With Multiple Highly Correlated Exposures and a Censored Survival Outcome. First Application in Ionizing Radiation Epidemiology

**DOI:** 10.3389/fpubh.2020.557006

**Published:** 2020-10-27

**Authors:** Marion Belloni, Olivier Laurent, Chantal Guihenneuc, Sophie Ancelet

**Affiliations:** ^1^PSE-SANTE/SESANE/LEPID, Institut de Radioprotection et de Sûreté Nucléaire, Paris, France; ^2^Université de Paris, Unité de Recherche “Biostatistique, Traitement et Modélisation des données biologiques” BioSTM - UR 7537, Paris, France

**Keywords:** Bayesian inference, ionizing radiation, lung cancer, multicollinearity, profile regression, survival data, truncated Dirichlet process mixture

## Abstract

As multifactorial and chronic diseases, cancers are among these pathologies for which the exposome concept is essential to gain more insight into the associated etiology and, ultimately, lead to better primary prevention strategies for public health. Indeed, cancers result from the combined influence of many genetic, environmental and behavioral stressors that may occur simultaneously and interact. It is thus important to properly account for multifactorial exposure patterns when estimating specific cancer risks at individual or population level. Nevertheless, the risk factors, especially environmental, are still too often considered in isolation in epidemiological studies. Moreover, major statistical difficulties occur when exposures to several factors are highly correlated due, for instance, to common sources shared by several pollutants. Suitable statistical methods must then be used to deal with these multicollinearity issues. In this work, we focused on the specific problem of estimating a disease risk from highly correlated environmental exposure covariates and a censored survival outcome. We extended Bayesian profile regression mixture (PRM) models to this context by assuming an instantaneous excess hazard ratio disease sub-model. The proposed hierarchical model incorporates an underlying truncated Dirichlet process mixture as an attribution sub-model. A specific adaptive Metropolis-Within-Gibbs algorithm—including label switching moves—was implemented to infer the model. This allows simultaneously clustering individuals with similar risks and similar exposure characteristics and estimating the associated risk for each group. Our Bayesian PRM model was applied to the estimation of the risk of death by lung cancer in a cohort of French uranium miners who were chronically and occupationally exposed to multiple and correlated sources of ionizing radiation. Several groups of uranium miners with high risk and low risk of death by lung cancer were identified and characterized by specific exposure profiles. Interestingly, our case study illustrates a limit of MCMC algorithms to fit full Bayesian PRM models even if the updating schemes for the cluster labels incorporate label-switching moves. Then, although this paper shows that Bayesian PRM models are promising tools for exposome research, it also opens new avenues for methodological research in this class of probabilistic models.

## 1. Introduction

Over the last decade, the human exposome has emerged as a novel and promising research paradigm in epidemiology, biomedical, and environmental health sciences (1–3). Originally proposed by Dr. Christopher Wild in 2005 (4), it encompasses the totality of human environmental (meaning all non-genetic) exposures throughout life—from conception to death. This concept, that argues for a holistic and integrated consideration of all environmental exposures simultaneously (5, 6), is the key complement to the genome in terms of understanding human health. Its initial aim is to decipher how complex environmental exposure situations lead to disease development. Its final aims are to gain more insight into the etiology of multifactorial and chronic pathologies, and, ultimately, to lead to better primary prevention strategies for public health. Obviously, cancers are among these pathologies for which the exposome concept is essential, as they result from the combined influence of many genetic, environmental (i.e., physical, biological, chemical) and behavioral stressors that may occur simultaneously and interact (7–11).

In epidemiological studies, it is thus important to properly account for multifactorial exposure patterns when estimating (or predicting) specific cancer risks at individual or population level. However, historically, epidemiological studies linking the adverse effects of environmental stressors and human health have mostly focused on characterizing the effect of a single stressor. This one is typically considered of “main interest” for investigation (12, 13). A few additional risk factors, including other environmental stressors, are usually considered, but this is most frequently because of their feared role as potential confounders. They are therefore adjusted for in regression models, in order to estimate the effect of the “main environmental stressor of interest” but independently from the potential influence of the other risk factors (14, 15). Only a few studies aim to estimate the interaction between exposure to an environmental stressor and other risk factors (e.g., smoking and asbestos or radon) (16, 17), and, even more rarely, the joint effects of exposure to several environmental stressors (e.g., ambient particles and ozone) (18). In the specific field of protection against the effects of ionizing radiation—that will be of interest in this paper—estimating radiation-related cancer risks and its uncertainty has been a key objective for decades, for the purpose of setting exposure limits (19). However, although ionizing radiation epidemiology has successfully reached that goal, the question of estimating how simultaneous environmental exposures to multiple radiological stressors of different nature potentially affect cancer risks has not yet been investigated thoroughly (20).

Estimating cancer risks due to simultaneous exposures to multiple environmental stressors may be challenging for several reasons, which are detailed elsewhere (21, 22). Particularly, major statistical difficulties occur when exposure-based risk factors are highly correlated. This occurs when collecting data on multiple environmental stressors during life. This may be also the case, for instance, when a worker is simultaneously exposed to many chemical and physical stressors in the course of his occupational activity. This situation will be referred to as co-exposure in the following. In this context, it is well-recognized that applying standard multiple regression models—in which at least two highly correlated predictors are assessed simultaneously- may lead to unstable risk coefficient estimates with high variance. Therefore, this approach may lead to misleading conclusions and unrealistic interpretations about the effect of each of the collinear predictors on the outcome variable (23–25). More sophisticated statistical methods must then be used to deal with this multicollinearity issue.

Although not yet widely used in practice (26), several statistical methods have been proposed to deal with multicollinearity and then, to potentially investigate the combined effect on health outcomes of highly correlated environmental stressors. Many previous studies relied on an environment-wide association approach (EWAS) where, in its simplest version, the association between each single exposure factor and the outcome was estimated separately (27, 28). Even if potentially useful to discover priority risk factors, this approach is mainly considered in an exploratory research phase and leads to limited investigations of an health-exposome association. Other approaches that have been proposed in this specific context mainly rely on: (a) variable selection in a regression context using, for instance, the elastic net criterion (29) or the Graphical Unit Evolutionary Stochastic Search (30); (b) data-driven dimension reduction using regression on principal components (31) or the sparse partial least squares regression (32, 33); (c) machine learning algorithms like recursive partitioning using random forests (34); and (d) clustering approaches to profile multiple correlated data (35) like k-means, the latent class analysis (LCA) (36) and the Bayesian profile regression mixture (PRM) models (37). Variable selection approaches are very interesting tools to identify a small subset of environmental stressors that are the “true villain” most responsible for affecting the health outcome of interest. They are particularly adapted when a huge number of stressors is considered. However, when only a few highly correlated exposure covariates are available, the idea is not to omit some of them in the study but rather to estimate an exposure-risk relationship using all available covariates and appropriate statistical methods to deal with multicollinearity issues. They may also be limited in their ability to efficiently differentiate true predictors from correlated covariates when the latter are very highly correlated (38). Data-driven dimension reduction aims at constructing summary latent variables as linear combinations of the original exposure covariates and then, to include these new uncorrelated variables in a multiple regression model (39). One major drawback is that these variables are constructed without considering the disease outcome of interest in principal component regression (PCR). Even if the sPLS (32) corrects for this by constructing uncorrelated latent variables as linear combinations of the original covariates and response variables, another drawback of data-driven dimension reduction approaches concerns the uncertainties related to this construction. Indeed, given that the disease risks are estimated in a second disjoint step, the loss of information about the uncertainty associated to this construction may lead to misleading interpretation of risk estimates. Finally, machine learning algorithms are both relevant and efficient approaches to deal with a huge number of stressors.

In this work, we focused on the specific problem of estimating the combined health effect—in terms of disease excess risk—of a few but highly correlated environmental exposure covariates, from a censored survival outcome. We opted for the PRM models. They are infinite mixture models that link a disease outcome to a set of correlated covariates through cluster membership. They are based on a Dirichlet process mixture as an attribution sub-model. By capturing the heterogeneity among the covariates, the PRM models allow both identifying specific patterns of covariate values—called covariate profiles—that are representative of a subpopulation (i.e., a cluster) and associating them with the disease outcome via a regression model. Then, inferring this probabilistic model allows both simultaneously identifying fine exposure profiles based on several correlated covariates, clustering individuals with similar risks and similar exposure characteristics and estimating the associated risk for each cluster. This joint modeling approach allows to rigorously capture uncertainty on all estimated parameters included in the different submodels. Compared to LCA and k-means algorithm, one of the principal motivations for PRM models is that the disease outcome influences cluster membership so that they can inform each other. Thus, the disease outcome may guide inference toward the most relevant clustering structures and is not only used during post-treatments. Another motivation for PRM models is that the number of clusters is unknown and informed by the data. Moreover, fitting PRM models under the Bayesian paradigm offers additional advantages. First, it allows dealing with the numerous latent variables included in these complex models and getting probabilistic answers to the studied question. Second, all uncertainty, including uncertainty associated with the clustering of the individuals, is reflected in credible intervals of risk parameters. Third, it provides the possibility to include external information on parameters in the form of prior distributions which is particularly useful when some unknown quantities of interest are not or only poorly informed by the data. Finally, it allows predicting the disease risk of a multi-exposed individual while conserving the uncertainty of estimated parameters. These models have already been employed in a variety of fields including genetics (40), environmental epidemiology (37, 41–44) and occupational epidemiology (45, 46) but never in ionizing radiation epidemiology. Note that an R package called PReMiuM (47) implements the Bayesian inference of PRM models for Gaussian, binary, ordinal, categorical, Poisson, and censored survival outcomes based on a Weibull distribution.

We extended the class of PRM models to deal with a censored survival outcome following an instantaneous excess hazard ratio model. This class of survival models is commonly used to estimate cancer risks in ionizing radiation epidemiology (48) but is not implemented in the PreMiuM package. The Bayesian inference of the proposed PRM model is conducted with a specific adaptive Metropolis-Within-Gibbs algorithm, implemented in Python and including three label switching moves. To illustrate our point, we applied our PRM model to the specific problem of estimating the risk of death by lung cancer among multi-exposed French uranium miners. Indeed, in the context of their work, underground uranium miners are simultaneously exposed to radon, external γ-ray and uranium dust (as well as other chemical and physical agents). Interestingly, these three sources of radiation exposure are highly correlated to each other in the French cohort of uranium miners. Actually, they are associated with the same initial phenomenon of disintegration of uranium, which is ubiquitous in uranium mines (49). Moreover, at this stage, an additive or synergic effect of co-exposure to these various radiological components on lung cancer risks cannot be excluded. Until now, most of the epidemiological studies on the French cohort of uranium miners have focused on studying the association between a chronic and low-dose exposure to radon and lung cancer mortality, as if radon—that is considered to be the second leading cause of lung cancer after smoking (50)—had an isolated effect. An EWAS approach was performed where the association between each single source of ionizing radiation and the risk of death by lung cancer was estimated separately, using a Poisson regression model. It showed that each source of ionizing radiation was significantly associated to a higher risk of death by lung cancer, in the French cohort of uranium miners (28). We propose to treat the multicollinearity issue in this case study, using our proposed Bayesian PRM model. Up to our knowledge, this is the first application of Bayesian PRM models to deal with highly correlated co-exposure in ionizing radiation epidemiology.

## 2. Materials and Methods

### 2.1. Study Population

The French cohort of uranium miners is a retrospective cohort whose characteristics, sources of data and methods of data collection (e.g., vital status, causes of death, …) have been described previously (28). Briefly, the last update included 5,086 males who were employed as uranium miners for at least 1 year in the CEA-COGEMA group between 1946 and 1990 and who were followed from 1946 to December 31, 2007. Uranium miners are simultaneously exposed to three sources of ionizing radiation: radon and its short-lived decay products (simply called radon hereafter), external γ-ray and uranium dusts. In the French cohort of uranium miners, the annual exposures to radon were assessed from 1946. On the other hand, the routine recording of occupational annual exposures to external γ-ray and uranium dust only began in 1956 in the French mines, following the introduction of radiation protection measures like the introduction of forced ventilation. In this paper, the study population was thus restricted to the so-called post-55 subcohort, in order to have simultaneous exposure measurements for the three sources of ionizing radiation. This subcohort included 3,377 miners from the original cohort who were first employed after December 31, 1955. At the end of follow-up, 94 miners had died of lung cancer. An age limitation of 85 years for follow-up was fixed due to the imprecision in determining the exact cause of death in those occurring after the 85th birthday (28). Main characteristics of the post-55 subcohort are recorded in Table 1.

**Table 1:** Main characteristics of the post-55 French cohort of uranium miners.

No. of miners	3,377
Age at entry into study, mean [min, max]	28.3 [16.9, 57.7]
Duration of work in years, mean [min, max]	16.7 [1.0, 40.9]
Duration of follow-up in years, mean [min, max]	32.8 [0.1, 51.0]
**Vital status**, ***n*** **(%)**	
Alive <85 years old	2,412 (71.4)
Alive ≥85 years old	74 (2.2)
Death from lung cancer	94 (2.8)
Death from another cause	777 (23.0)
Lost to follow-up	20 (0.6)
Exposure to radon^*^	
Exposed miners, *n* (%)	2,910 (86.2)
Duration of exposure (in years), mean [min, max]	12.9 [1.0, 35.0]
Cumulative exposure (in WLM), mean [min, max]	17.8 [0.003, 128.4]
**Exposure to** γ-rays^*^	
Exposed miners, *n* (%)	3,240 (95.9)
Duration of exposure (in years), mean [min, max]	13.2 [1.0, 36.0]
Cumulative exposure (in mSv), mean [min, max]	54.9 [0.2, 470.1]
Exposure to uranium dusts^*^	
Exposed miners, *n* (%)	2,746 (81.3)
Duration of exposure (in years), mean [min, max]	12.9 [1.0-35.0]
Cumulative exposure (in kBq·m^−3^·h), mean [min, max]	1.64 [0.01, 10.4]

* *Results only on measured exposures*.

### 2.2. Multiple Exposure Assessment, Proxy Variables, and Multicollinearity

In the French cohort of uranium miners, information on radon, external γ-ray exposures and uranium dusts exposure was assessed individually for each year of employment, but the method of measurement changed over time. Between 1946 and 1955, there was no systematic exposure assessment in the French uranium mines. Therefore, the annual radon exposure, expressed in working level months (WLM), was retrospectively reconstructed by a group of experts for this period, based on environmental measurements performed in the mines and information concerning the miners' type of work and location. Then, from 1956, the individual radon exposure was recorded systematically, following the new radiation protection measures which were set up at this date. More specifically, from 1956 to 1982, individual radon exposure was assessed from monthly ambient concentration measurements and information about the miners' activity (i.e., job type, location, and time spent at each location). From 1983, annual radon exposure was individually recorded, using personal dosimeters integrated to the Individual System of Integrated Dosimetry (ISID). Personal dose equivalents due to γ-ray exposures, expressed in millisieverts (mSv), were recorded individually since 1956, using two different types of personal dosimeters, depending on the calendar period: personal film badge dosimeters (CEA PS1 type) from 1956 to 1985 and personal thermoluminescence dosimeters (TLDs) integrated to the ISID from 1986 onwards. Finally, the annual exposure to long-lived radionuclides arising from uranium ore dust, expressed in Becquerels per cubic meter hour (Bq·m^−3^·h), was retrospectively reconstructed for the period 1956–1958 (51). It was then assessed from monthly ambient measurements from 1959 to 1982. From 1983, individual measurements were collected with the ISID.

Potentially relevant proxy variables are also available in the French cohort of uranium miners to reflect the uranium miners' working conditions and any other occupational exposures. First, there are the job types of French uranium miners which are classified into five categories : (1) hewers before mechanization, (2) hewers after mechanization, (3) other underground work before mechanization, (4) other underground work after mechanization, and (5) surface worker. The mechanization of work in the French uranium mines began in 1977, with the introduction of trucks. Thus, from 1977 onwards, the uranium miners' working conditions can be assumed to be less physically demanding compared to the period before mechanization. But on the other hand, an additional occupational exposure to diesel, recognized as a lung carcinogen (52), appeared in the mines at the same period. Finally, hewers were assumed to have a more physically demanding labor and harsher working conditions than other underground and open-pit uranium miners. An additional proxy for uranium miners' working conditions is the working location which includes four different mining districts: (1) Vendée, (2) Crouzille, (3) Forez, and (4) Hérault. Actually, the type of uranium deposits (i.e., granitic, sedimentary) has an impact on the undergrounds galleries of the uranium mines and then, on the miners' working conditions. Note that the type of uranium deposits depends on the mining district. It is granitic for the districts of Vendée, Crouzille, and Forez and sedimentary for the district of Hérault (53).

**Figure 1:**
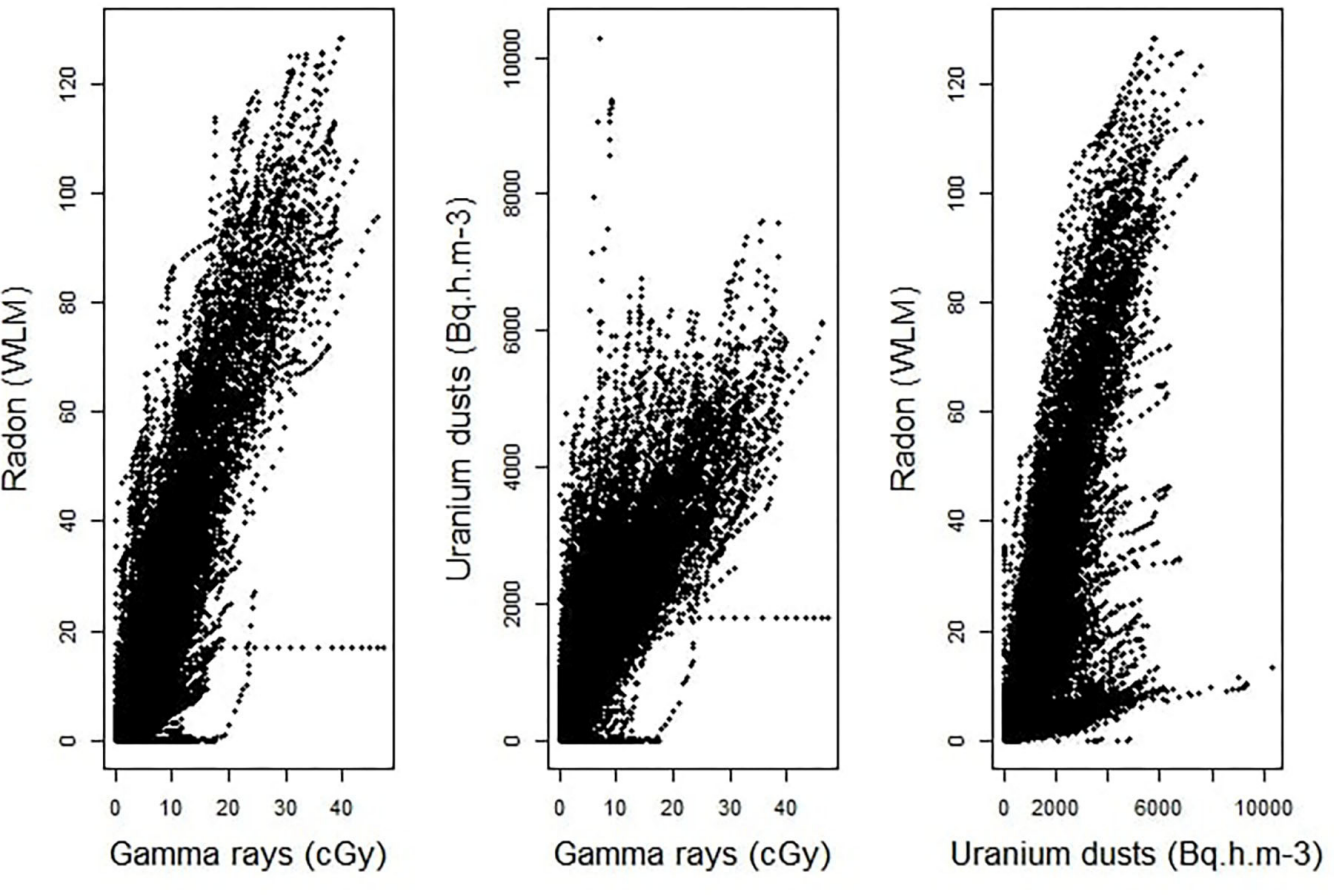
Scatter plots of the observed cumulative exposures to γ-rays and radon (left-hand panel), γ-rays and uranium dust (at the center), radon and uranium dust (right-hand panel).

An estimation of Pearson's correlation coefficients, using all the available pairs of cumulative exposures to two different sources of ionizing radiation, clearly shows that the assessed values of occupational exposures to radon, γ-ray and uranium dusts are highly correlated in the post-55 subcohort of French uranium miners. Indeed, the estimated coefficients are pretty high. It is equal to 0.90 between radon and γ-ray, to 0.82 between uranium dusts and γ-ray and to 0.78 between radon and uranium dusts. Figure 1 displays the scatter plots of the observed cumulative exposures to the three sources of ionizing radiation. It clearly confirms that we are faced with a multicollinearity issue, requiring the use of a suitable statistical approach to estimate the combined effect of these three radiological exposures, the job type and the localization of the mine on the risk of death by lung cancer in the post-55 subcohort of French uranium miners.

### 2.3. Model Formulation

To deal with multicollinearity in the specific context of estimating the combined effect of a few but highly correlated exposure covariates, we opted for a Bayesian profile regression mixture PRM model. In this approach, three submodels must be specified and linked, through conditional independence assumptions: the disease, the exposure and the attribution submodels. A Bayesian PRM model is a hierarchical model that allows jointly describing: (a) the association between a disease outcome (e.g., the age at death by lung cancer of a miner) and an exposure profile (disease sub-model); (b) the probability distribution of the different covariates of interest in each cluster, in order to characterize specific exposure profiles (exposure sub-model); and (c) the random assignment of an individual to a given profile (or cluster) (attribution sub-model).

*The disease sub-model* conventionally used in radiation epidemiology is an Excess Hazard Ratio (EHR) model. Let *S*_*i*_ be the age (in days) at death by lung cancer of miner *i*, *i* ∈ 1, 2, ..., *n* where *n* is the total number of miners. Let *R*_*i*_ be the right-censored age defined as the earliest age of miner *i* among age at death by a cause other than lung cancer; age on December 31, 2007; age in days corresponding to his 85th birthday and age until loss to follow up. For each miner *i*, the observed outcome of interest can therefore be represented by the non-negative continuous variable *Y*_*i*_ = *min*(*S*_*i*_, *R*_*i*_) and the binary variable δ_*i*_ where δ_*i*_ = 1 if *S*_*i*_ ≤ *R*_*i*_ (i.e., miner *i* died of lung cancer at age *Y*_*i*_ = *S*_*i*_) and δ_*i*_ = 0 if *S*_*i*_ > *R*_*i*_ (i.e., miner *i* “would have died of lung cancer” after age *R*_*i*_). The instantaneous hazard rate of death by lung cancer of miner *i* at age *t*, noted *h*_*i*_(*t*) is defined by

(1) $$\begin{array}{l} h_{i}\left(t\right)=h_{0}\left(t\right)\cdot \left(1+\beta_{C_{i}}\right) \end{array}$$

Baseline hazard *h*_0_(*t*) is the instantaneous risk of death by lung cancer at age *t* by not exposed profile (the reference cluster of miners not exposed to ionizing radiation), *C*_*i*_ is the cluster label of miner *i* and β_*c*_ is the instantaneous excess risk of death by lung cancer of the cluster *c*. Thus, two miners belonging to the same cluster *c* have the same risk of death by lung cancer. Note that ∀*c*, β_*c*_ is subject to the constraint β_*c*_ > −1 to ensure the positivity of *h*_*i*_(*t*).

Following Hoffmann et al. (48), *h*_0_(*t*) is assumed to be piece-wise constant on four age intervals for which values of baseline hazard are assumed to be constant. These four intervals correspond to a partition of age axis defined by before 40 years old, between 40 and 55, between 55 and 70 and finally after 70 years old. The corresponding four constants of baseline hazard are denoted by λ_1_, λ_2_, λ_3_ and λ_4_.

When modeling lung cancer mortality in the French cohort of uranium miners, we considered the age at death by lung cancer of each miner as disease outcome. Indeed, Kleinbaum suggested to favor age as time-scale whenever age at event is likely to have a larger effect on the hazard than time-on-study (54). Moreover, based on previous findings on cohorts of uranium miners, we can assume that, contrary to the attained age of a miner, the timing of study initiation has no inherent meaning in terms of the risk of lung cancer mortality in the cohort. Finally, several authors recommend to favor age as time-scale whenever possible since the modeling of the effect of age can be complex and prone to misspecification errors. Based on these arguments, we chose attained age as time scale. Thus, age is still accounted for in the disease model.

*The exposure sub-model* defines clusters based on covariates levels and on a similar risk to lung cancer death. Probability distribution of the covariates conditionally to a cluster is introduced. The different covariates considered for clusters include cumulative radiation exposures and other characteristics of miners. Details on these covariates are the following:

- Cumulative exposure of occupational radon $X_{i}^{R}$, γ-rays $X_{i}^{G}$, and uranium dust $X_{i}^{D}$ during the whole following up period of miner *i*;
- Job type *J*_*i*_ most occupied by miner *i*. This categorical variable have five modalities: (1) hewers before mechanization, (2) hewers after mechanization, (3) other underground work before mechanization, (4) other underground work after mechanization, and (5) surface work;
- Age at first exposure *A*_*i*_ of miner *i*. Sensibility of radiation can be function of age of exposure (55);
- Localization of the mine *M*_*i*_. We distinguished Hérault mine and the others based on the deposit's type;
- Exposure duration *T*_*i*_ of the miner *i*. Four duration periods with similar number of miners are considered: miners who were exposed 5 years and less, 6–12 years, 13–18 years, and finally those who have been exposed for at least 19 years.

The probability distribution of each covariate depends on parameters which are function of the cluster *c*. We assumed lognormal distributions LogN($\mu_{c}^{X},\sigma_{c}^{X}$) for positive and continuous variables and multinomial distributions Multinomial($p_{c}^{X}$) for categorical variables.

The different distributions are the following:

(2) $$\begin{array}{l} \{\begin{array}{ll} X_{i}^{R}|C_{i}=c,\mu_{c}^{R},\sigma_{c}^{R} & \sim \;LogN\left(\mu_{c}^{R},\sigma_{c}^{R}\right)\\ X_{i}^{G}|C_{i}=c,\mu_{c}^{G},\sigma_{c}^{G} & \sim \;LogN\left(\mu_{c}^{G},\sigma_{c}^{G}\right)\\ X_{i}^{P}|C_{i}=c,\mu_{c}^{P},\sigma_{c}^{P} & \sim \;LogN\left(\mu_{c}^{P},\sigma_{c}^{P}\right)\\ \;A_{i}|C_{i}=c,\mu_{c}^{A},\sigma_{c}^{A} & \sim \;LogN\left(\mu_{c}^{A},\sigma_{c}^{A}\right)\\ \;J_{i}|C_{i}=c,p_{c}^{J} & \sim \;Multinomial\left(p_{c}^{J}\right)\\ M_{i}|C_{i}=c,p_{c}^{M} & \sim \;Multinomial\left(p_{c}^{M}\right)\\ \;T_{i}|C_{i}=c,p_{c}^{T} & \sim \;Multinomial\left(p_{c}^{T}\right) \end{array} \end{array}$$

*The attribution sub-model* associates miner *i* to a cluster *C*_*i*_ based on the probability ϕ_*c*_ of belonging to the cluster *c*. Let *C*_*max*_ be the maximum number of clusters, ϕ = (ϕ_1_, ϕ_2_, ..., ϕ_*C*_*max*__) defines the vector of the probabilities of assignment to each cluster among the *C*_*max*_ ones. The parameter vector ϕ follows a Dirichlet process. Due to the Dirichlet process, the number of non-empty groups is not arbitrarily fixed but estimated, only the maximum number of clusters *C*_*max*_ is given. The construction of these mixing weights ϕ = (ϕ_1_, ϕ_2_, ..., ϕ_*C*_*max*__), also called “stick-breaking,” is the following:

(3) $$\begin{array}{l} V_{c}\sim Beta\left(1,\alpha \right),c\in \left\{1,...,C_{max}-1\right\} \end{array}$$

(4) $$\begin{array}{l} \phi_{c}=V_{c}\cdot \left(1-\overset{c-1}{\underset{k=1}{\sum }}\phi_{k}\right),c\in \left\{1,...,C_{max}-1\right\} \end{array}$$

(5) $$\begin{array}{l} \phi_{C_{max}}=1-\overset{C_{max}-1}{\underset{k=1}{\sum }}\phi_{k} \end{array}$$

The number of non-empty clusters is guided by α. A small value of α reduces the probability to have a large number of non-empty clusters, and respectively. This “stick-breaking” construction approximates the infinite cluster model with a finite one. The value of *C*_*max*_ has to be chosen large enough to give a good approximation but small enough to avoid unnecessary calculations. *C*_*max*_ should be set so that the probability ϕ_*C*_*max*__ is expected to be small (56). The choice of *C*_*max*_ is highly affected by the value of α, and for α up to 10, the probability ϕ_*C*_*max*__ is negligible with *C*_*max*_ equals to 50 (57). Some guidelines and more detailed description are given in Molitor et al. (37).

### 2.4. Prior Distributions and Bayesian Inference

#### 2.4.1. Prior Distributions

Prior distributions are chosen poorly informative except for parameters involved in baseline hazard, in stick-breaking prior as well as means of exposure for which external information were available.

Thus, normal centered distributions with large variance were considered for the risk parameters β_*c*_ and for the means of age at first exposure $\mu_{c}^{A}$ (on log scale) in each group *c, c* = 1, ..., *C*_*max*_. Large Uniform distributions were considered for the geometric standard deviation parameters of the lognormal distributions $\sigma_{c}^{R}$, $\sigma_{c}^{G}$, $\sigma_{c}^{P}$, and $\sigma_{c}^{A}$. Dirichlet prior distributions with parameters equal to 1/2 were considered for the parameters of multinomial distributions, namely $p_{c}^{J}$, $p_{c}^{M}$, and $p_{c}^{T}$.

Concerning the mean of γ-rays $\mu_{c}^{G}$, radon $\mu_{c}^{R}$, and uranium dust $\mu_{c}^{P}$ exposures (on log scale), information are available from German uranium miner cohort (58). Normal prior were considered for $\mu_{c}^{G}$, $\mu_{c}^{R}$ and $\mu_{c}^{P}$ with means and variances based on exposure levels of this cohort.

As parameters involved in baseline hazard are poorly informed by data in particular for young miners, external data on lung cancer mortality among men in France between 1968 and 2005 were used to specify the informative prior gamma distributions on the parameters λ_1_, λ_2_, λ_3_, and λ_4_ defining the baseline risk of death by lung cancer among French uranium miners (assumed constant by age intervals). Finally, as recommended by Molitor et al. (37), we used a uniform distribution on the interval [0.3, 10] for the parameter α which influences the number of non-empty clusters *a posteriori*. All details are given in Table 2.

**Table 2:** Prior probability distributions assigned to the unknown parameters of a Bayesian PRM model including the disease sub-model, the exposure sub-model and the attribution sub-model.

	**Parameter**	**Family**	
Disease sub-model	β_*C*_	Normal	N (0, 10^6^)
	λ_1_	Gamma	G (23.7, 4.9·10^8^)
	λ_2_	Gamma	G (35.5, 2.6·10^7^)
	λ_3_	Gamma	G (88.1, 1.6·10^7^)
	λ_4_	Gamma	G (29.7, 3.2·10^6^)
Exposure sub-model	$\mu_{c}^{G}$	Normal	N (0.10, 2.25)
	$\mu_{c}^{R}$	Normal	N (−2.3, 8.08)
	$\mu_{c}^{P}$	Normal	N (1.01, 11.79)
	$\mu_{c}^{A}$	Normal	N (0, 10^6^)
	$\sigma_{c}^{G},\sigma_{c}^{R},\sigma_{c}^{P},\sigma_{c}^{A}$	Uniform	U [0,100]
	$p_{c}^{J},p_{c}^{M},p_{c}^{T}$	Dirichlet	D [0.5, …, 0.5]
Attribution sub-model	α	Uniform	U [0.3, 10]

#### 2.4.2. Bayesian Inference

**Figure 2:**
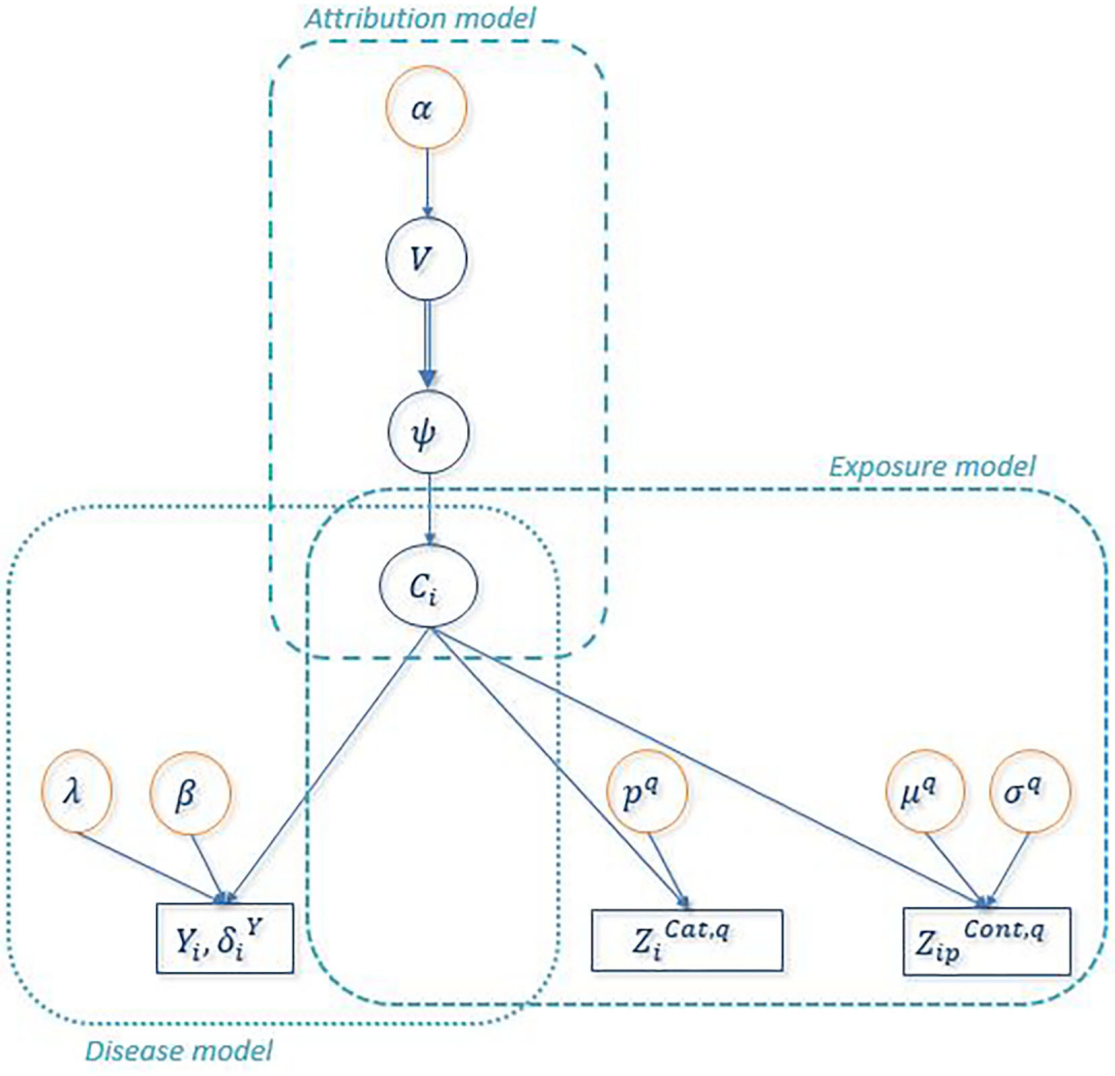
Directed Acyclic Graph associated to the full Bayesian PRM model. Circles indicate unknown quantities and rectangles indicate observed variables. Single arrows indicate oriented probabilistic links between two quantities and double arrows indicate oriented deterministic links between two quantities. $Z_{i}^{Cat,q}$ denotes the observed value of any categorical covariate *q* for uranium miner *i* and $Z_{i}^{Cont,q}$ denotes the observed value of any continuous covariate *q* of uranium miner *i*.

Figure 2 shows the directed acyclic graph for the full hierarchical model combining the disease sub-model, the exposure sub-model and the attribution sub-model. R package “PReMiuM” already exists to implement the Bayesian profile regression (47) for Bernoulli, Binomial, Poisson, Normal, categorical response as well as Weibull survival model. Unfortunately, the EHR survival model is not a possible option in this package. Thereby, a Markov Chain Monte Carlo (MCMC) algorithm was implemented in Python to sample from the joint posterior distribution of all unknown parameters and latent variables. Simulations were performed in order to validate the code, results of these simulations can be found in the Supplementary Material. We used a Metropolis-within-Gibbs algorithm (59) to conduct the Bayesian inference, as full conditional distributions were not always analytically tractable. An adaptive phase of Metropolis-Hastings steps, which is necessary to improve the convergence and the efficiency of the algorithm, updates the variance of each proposal distribution to target an acceptance rate of 40% for single parameters and 20% for vectors (59). The parameters and the latent variables were updated separately. We ran 100 steps of 100 iterations for the adaptive phase, then 10,000 iterations were dropped for the burn-in phase and finally 150,000 additional iterations were run. To decrease within-chain autocorrelations, we thinned the sample by storing only every 20 iterations. Posterior sample of each unknown quantity therefore contains 7,500 values. A particular attention was done on the convergence toward local modes by considering different initial values for parameter α directly linked to the number of non-empty clusters. Moreover, as suggested by Liverani et al. (47), we introduced three label switching moves in order to try to best avoid convergence to local mode (60, 61). The use of this three label switching moves is justified by the weak identifiably of the clusters labels leading to multiple modes of the posterior distributions of the ϕ_*c*_'s. To explore multimodal posterior distributions, Papaspiliopoulos and Roberts (60) introduce two label switching moves which allow moves particularly at the beginning of MCMC algorithm. To improve ability of moves, Hastie et al. (61) add a third one . The basic idea of moves is to switch two labels *j* and *k* according to a probability min (1,*r*_*jk*_). Details on *r*_*jk*_ are given in Table 3. Main characteristics of these three moves are the following. The first move has high acceptance probability of switching *j* and *k* when weights ϕ_*j*_ and ϕ_*k*_ are close. On the other hand, two clusters with similar number of miners are rarely switched. The two other moves propose only switch between two neighboring clusters namely *j* and *j* + 1. When label switching is accepted according to the second or third switching moves, the respective beta components V involved in the stick-breaking procedure are simultaneously modified (and consequently the weights ϕ). The second move corresponds to high acceptance probability for neighboring clusters including different number of miners. For the third label switching, the respective beta components V are modified so that the corresponding weights ϕ_*j*_ and ϕ_*j*+1_ are close to their expectation conditional on these new labels. Details on *r* and V are given in Table 3. For the three switching procedures, corresponding excess risks β and other cluster specific parameters are simply exchanged when move is accepted.

**Table 3:** Label switching moves.

	**Move 1**	**Move 2**	**Move 3**
*r*_*jk*_	${\left(\frac{\phi_{j}}{\phi_{k}}\right)}^{n_{k}-n_{j}}$	$\{\begin{array}{cc} \frac{{\left(1-V_{j+1}\right)}^{n_{j}}}{{\left(1-V_{j}\right)}^{n_{j+1}}} & k=j+1\\ 0 & \text{otherwise} \end{array}$	$\{\begin{array}{cc} {\left(\frac{\phi^{+}}{\phi_{c+1}R_{1}+\phi_{c}R_{2}}\right)}^{n_{j}+n_{j+1}}R_{1}^{n_{j+1}}R_{2}^{n_{j}} & k=j+1\\ 0 & \text{otherwise} \end{array}$
$V_{l}^{\prime }$	*V*_*l*_	$\{\begin{array}{ll} V_{j+1} & I=j\\ V_{j} & I=j+1\\ V_{I} & \text{otherwise} \end{array}$	$\{\begin{array}{cc} \frac{\phi_{j}^{\prime }}{\prod_{k<j}\left(1-V_{k}\right)} & l=j\\ \frac{\phi_{j+1}^{\prime }}{\left(1-V_{j}^{\prime }\right)\prod_{k<j}\left(1-V_{k}\right)} & l=j+1\\ V_{l} & \text{otherwise} \end{array}$

*The switching between labels j and k is accepted with probability min(1, r_jk_). If label switching is accepted, V′ is the new value of beta-component V. n_j_ is the number of miners in cluster j*.

$\phi^{+}=\phi_{j}+\phi_{k}$, $\phi^{\prime }=\phi_{j+\text{1}}\frac{E\left(\phi_{j}|C^{\prime },\alpha \right)}{E\left(\phi_{j+\text{1}}|C,\alpha \right)}+\phi_{j}\frac{E\left(\phi_{j+\text{1}}|C^{\prime },\alpha \right)}{E\left(\phi_{j}|C,\alpha \right)}$,

$R_{\text{1}}=\frac{\text{1}+\alpha +n_{j+\text{1}}+\underset{l>j+\text{1}}{\sum }n_{l}}{\alpha +n_{j+\text{1}}+\underset{l>j+\text{1}}{\sum }n_{l}}$ and $R_{\text{2}}=\frac{\alpha +n_{j}+\underset{l>j+\text{1}}{\sum }n_{l}}{\text{1}+\alpha +n_{j}+\underset{l>j+\text{1}}{\sum }n_{l}}$

### 2.5. Post-treatment

As described in Molitor et al. (37) and in Liverani et al. (47), the post-treatment is realized after running the MCMC algorithm. We chose to determine an optimal partition corresponding to a partition sampled from our MCMC algorithm. The main advantage of using a sampled partition is to avoid difficult problems linked to clusters labels which could be different between iterations. There are different techniques to obtain this optimal partition. We decided to use the post-processing approach based on a posterior similarity matrix. Another possibility could have been to use the MAP estimate corresponding to the partition leading to the highest value of the marginal posterior distribution. As mentioned by Liverani et al. (47), the MAP estimate is more sensitive to the Monte Carlo error than the techniques based on the similarity matrix. If *K* is the number of iterations, *K* binary square matrices *S*_*k*_ of dimension *n* × *n* are determined at each iteration *k* where *S*_*k*_(*i, j*) = 1 if miners *i* and *j* share the same cluster at iteration *k* of the MCMC sampler, and 0 if not. The mean *S* of these *K* matrices (*S*_1_, ..., *S*_*K*_) thus contains the proportion that two miners belong to the same cluster during MCMC sampler. The estimated best partition called *C*^*best*^ is the one that minimizes the least-squared distance to matrix *S*. *C*^*best*^ is a vector such that $c_{i}^{best}$ is equal to the cluster label of miner *i* in this optimal partition.

Posterior distributions of parameters are obtained conditionally to the best partition *C*^*best*^. Generally speaking, if θ_*c*_ denotes a parameter depending of cluster *c*, a sample from posterior distribution of parameter θ_*c*_ conditionally to partition *C*^*best*^ is {${\overset{-}{\theta }}_{c,k},k=1,...,K$} such that

(6) $$\begin{array}{l} {\overset{-}{\theta }}_{c,k}=\frac{1}{n_{c}}\underset{i:c_{i}^{best}=c}{\sum }\theta_{c_{i}^{k},k} \end{array}$$

with *n*_*c*_ the number of uranium miners in cluster *c* and $c_{i}^{k}$ the cluster of miner *i* at iteration *k*. This post-processing procedure is apply on all parameters depending on cluster label involved in the three sub-models that is (β, μ^*R*^, σ^*R*^, μ^*G*^, σ^*G*^, μ^*P*^, σ^*P*^, μ^*A*^, σ^*A*^, *p*^*J*^, *p*^*M*^, *p*^*T*^) as well as the weights ϕ of clusters.

## 3. Results

### 3.1. Univariate and Multivariate EHR Model Without Clustering

In order to assess impact of multicollinearity on EHR model, classical Excess Hazard Ratio model was implemented without clustering procedure. The instantaneous hazard rate of death by lung cancer of miner *i* at time *t* *h*_*i*_(*t*) is here directly function of exposures, without taking into account of multicollinearity. A first approach consists in considering each radiation source separately and secondly, to include simultaneously the three ones. Posterior median and 95% credible interval of β are obtained in each case. With only one exposure, *h*_*i*_(*t*) is then defined by *h*_*i*_(*t*) = *h*_0_(*t*) · (1 + β · *X*_*i*_) where baseline hazard *h*_0_ is assumed piece-wise constant as previously, β the excess risk of death by lung cancer associated to cumulative exposure X and *X*_*i*_ the cumulative exposure of miner *i*. When considering single exposure, X can be X^*R*^, X^*G*^ or X^*D*^ for respectively radon, γ-rays and uranium dust. Posterior medians of β and associated 95% credible intervals are 2.7 [1.1 , 5.2], 0.78 [0.28 , 1.67], and 3.34 10^−2^ [1.07 10^−2^ , 7.00 10^−2^] for respectively radon, γ-rays and uranium dust. As zero is excluded from each credible interval, the excess risk of death by lung cancer is strictly positive for each exposure. When considering simultaneously the three exposures of ionizing radiations, then $h_{i}\left(t\right)=h_{0}\left(t\right)\cdot \left(1+\beta_{R}X_{i}^{R}+\beta_{G}X_{i}^{G}+\beta_{D}X_{i}^{D}\right)$. Posterior medians of β_*R*_, β_*G*_ and β_*D*_ with associated credible intervals are now 2.7 [−0.2 , 5.8], 0.00 [−0.39 , 1.17], and −0.15 10^−2^ [−1.66 10^−2^, 3.81 10^−2^], respectively. None of the exposures were significantly associated to the risk of death by lung cancer anymore. This result highlights the issue of multicollinearity of the exposures in our case. When considering exposure one per one, the values of estimated risks are difficult to interpret because could also be due to confusing effect from the other radiation sources which are both correlated with death by lung cancer and with studied exposure. As expected, introduction of simultaneous exposures leads to huge imprecision and consequently to no significant associations for some radiological exposures.

### 3.2. Convergence Toward Local Mode Under PRM Model

**Figure 3:**
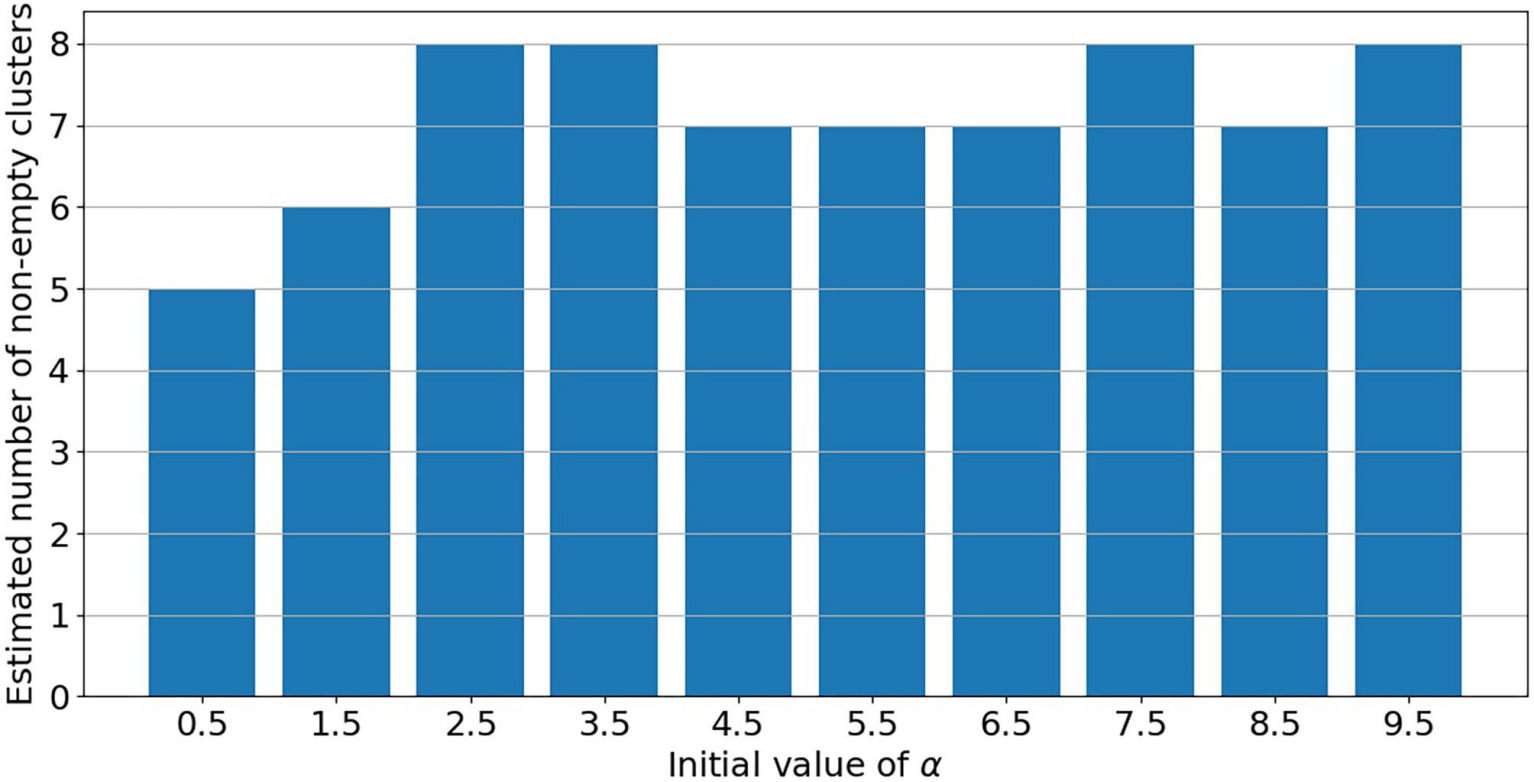
Estimated number of non-empty clusters according to the initial value of α.

PRM model as defined in section 2.3 is implemented on the post-55 sub-cohort. As already mentioned in Liverani et al. (47), parameter α in Equation (3) is directly linked to the number of non-empty clusters. Under PRM model, this number is also estimated (only the maximum number of clusters *C*_*max*_ is fixed) and a particular attention has to be made on local convergence issue even if label switching moves are introduced. To assess a convergence toward a local mode, MCMC samplers were run from different initial values of α. Initial values are chosen from 0.5 to 9.5 covering the prior support of α. For a given initial value, the number of non-empty clusters systematically converges to a single value without moves during the sampler, while there is no convergence issue for the other parameters. Results are presented in Figure 3 where the number of non-empty clusters takes four possible values from 5 to 8 (including the cluster of non-exposed uranium miners) according to the different initial values of α. Local convergence issue is also clearly suspected despite the three label switching procedure. An explanation could be the low proportion of miners died from lung cancer. This proportion is indeed near 3% giving a low signal to infer the risk between clusters and lung cancer. Consequently, a restricted profile regression mixture RPRM model is considered where the number K of non-empty clusters is fixed. The attribution sub-model defined section 2.3 is then simplified where the weights ϕ have now a fixed number K of component. We ran MCMC algorithm from two different sets of initial values. A solution to choose K could have been to choose one value among the four values suggested by Figure 3. Deviance information criteria (DIC) (62) as well as Watanabe-Akaike, also called Widely Applicable, Information Criterion (WAIC) (63) are presented in Table 4 for K from 5 to 8. These two criteria are concordant in favor of 8 non-empty clusters. As penalized deviance is well-known to possibly select most complex models, we prefer to present results with K equal to 8 non-empty clusters but also to compare with the three other RPRM models corresponding to 5, 6, or 7 non-empty clusters (results given in the Supplementary Material). Note that when the number of non-empty clusters is fixed, no convergence issue was found for all other parameters.

**Table 4:** DIC and WAIC of Bayesian PRM model according to the fixed number K of non empty clusters.

**Number K of non-empty clusters**	**DIC**	**WAIC**
5	146,345	110,872
6	136,714	108,773
7	118,602	107,004
8	104,566	105,704

### 3.3. Results With Fixed Number of Non-empty Clusters

**Figure 4:**
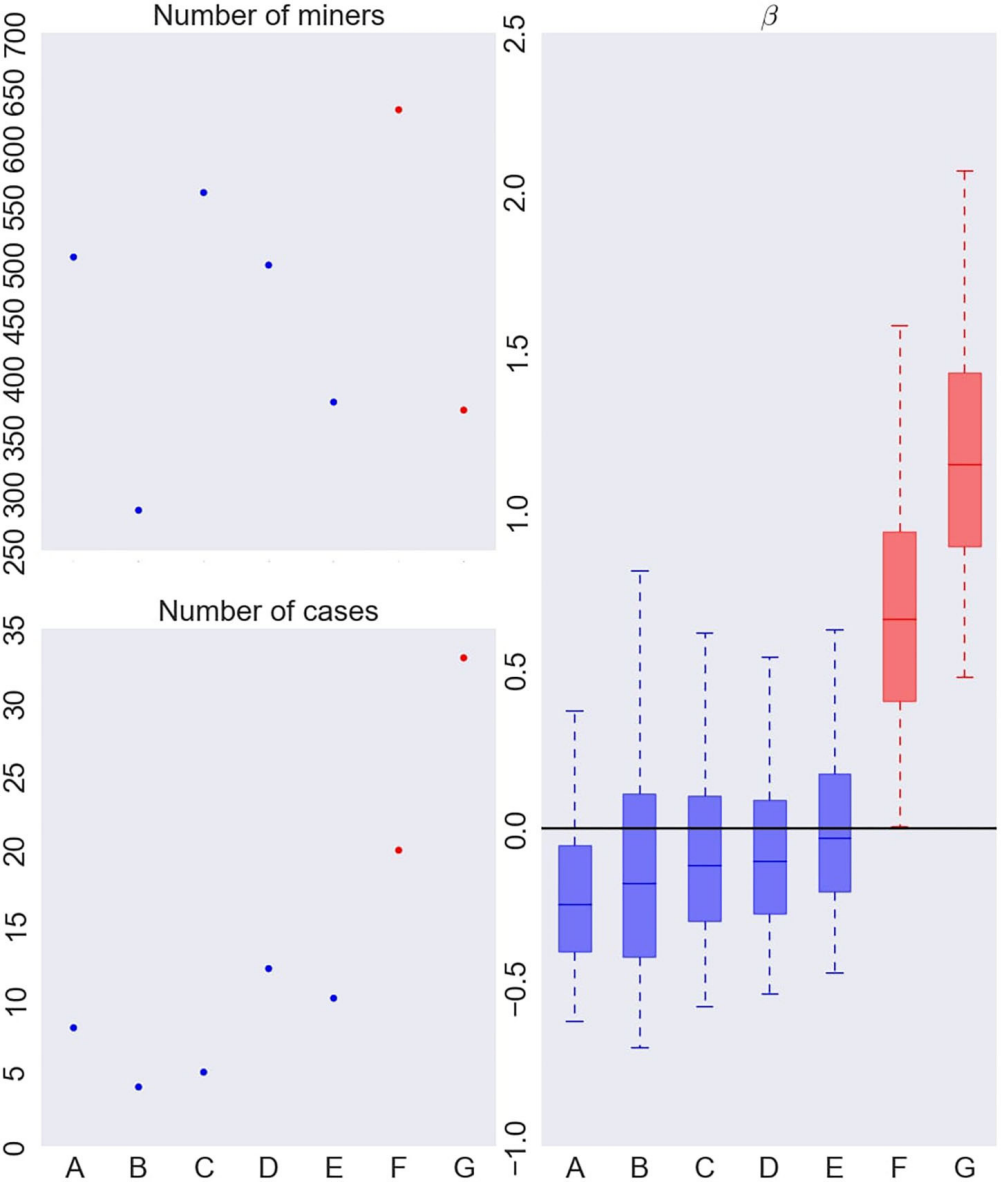
Number of French uranium miners (top left), number of deaths by lung cancer (bottom left) and instantaneous excess hazard ratio (per 100 WLM) of death by lung cancer (β) in each cluster (right), when fitting a Bayesian RPRM model assuming 8 non-empty clusters from the French cohort of uranium miners. The cluster including non-exposed miners is not displayed. The boxes represent the three quartiles (1st quartile, median, and 3rd quartile) of the posterior distribution of β and the whiskers of the boxplots show the 95% credible interval of the posterior distribution for each group.

**Figure 5:**
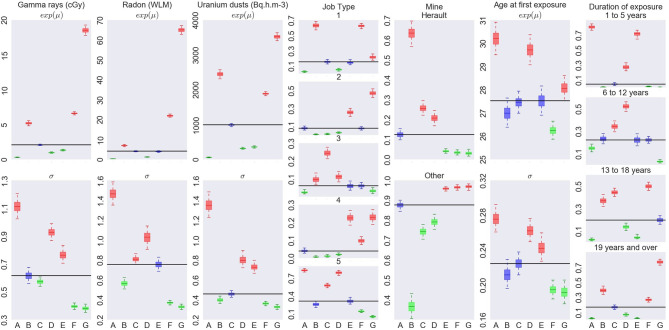
Characterization of the exposure profiles associated to each cluster, when fitting a Bayesian RPRM model assuming 8 non-empty clusters. The cluster including non-exposed miners is not displayed.

Results for eight clusters model are summarized on Figures 4, 5 while results for the 5–7 clusters can be found in the Supplementary Material.

On the left of Figure 4, number of miners (top) and number of cases (bottom) per cluster are represented except for the cluster of non-exposed uranium miners. The seven resulting clusters are denoted by A to G. The order of clusters representation follows the order of the associated estimated risk of each cluster. Thus, cluster A corresponds to the lowest estimated posterior median of β and cluster G to the higher one. The number of miners varies from 285 to 633 and the number of cases per cluster from 4 to 30. On the right of Figure 4, results on the excess risk of death by lung cancer of each cluster (β_*A*_ to β_*G*_) are given. Boxes correspond to the posterior quartiles of β and the whiskers extend to the posterior 2.5% and 97.5% quantiles illustrating 95% credible interval of β. Colors indicate whether posterior 95% credible interval of β is greater than zero (red) or include zero (blue). A cluster is called “significant high risk cluster” (or respectively “significant low risk cluster”) if whiskers are >0 (respectively lower than 0). Two significant high risk clusters are here identified, namely clusters F and G. The posterior median excess risk of cluster G is estimated to 1.14 and to 0.66 for cluster F. Note that an excess risk of 1.14 means that miners belonging to this cluster have a risk multiplied by 2.14 compared to non-exposed uranium miners.

Characterization of each cluster in terms of covariates is illustrated on Figure 5. Each column corresponds to one covariate, cluster labels being specify on horizontal axis. For continuous covariates, such as cumulative exposures and age at first exposure, results on medians (*e*^μ^) are on the top while results on standard deviation (on log scale) on bottom. For categorical covariates, such as Job type, Mine and Exposure duration, posterior distribution of probability of each category is shown. Boxes and whiskers are defined as previously. The two different colors, green and red, correspond to a 95% credible interval, respectively under or upper the global median on all miners (whatever the cluster) while blue color shows no particular values of the covariate for this cluster.

The cluster G with the highest risk of death by lung cancer corresponds to the most exposed uranium miners as credible intervals of the mean for cumulative radon, γ-rays and uranium dust exposure are high. They were mainly working before mechanization or as hewer after mechanization, not in Herault's mine, pretty old when they started working compared to the other groups and being exposed during long time (longer than 19 years). This cluster corresponds to the most difficult working conditions. This high risk cluster is found for 5, 6, or 7 non-empty clusters (see Supplementary Material). Its systematic identification is reassuring in terms of model validity since it is consistent with standard assumptions in the field.

The cluster F associated to the second highest risk of death by lung cancer is characterized by miners who were also highly exposed but less than in cluster G, worked as hewer after mechanization or other underground job before mechanization, not working in Hérault's mine, were young when they started working compared to the other groups and exposed more than 13 years. Working conditions of this second cluster can also be considered as difficult but less than those of cluster G in particular concerning hewer before or after mechanization and the duration of exposure a little lower. On the other hand, this second cluster highlights risk profile of miners who started to work early compared to the other groups. Results concerning this second cluster differ slightly depending on the fixed number of non-empty clusters (see Supplementary Material). Indeed, this cluster is associated to a positive excess risk which is significant for RPRM with K = 7, nearly significant with K = 6 but not significant with K = 5. Posterior medians of β_*F*_ and β_*G*_ as well as characteristics of these two clusters are very similar with K = 6 and K = 7 to those already found with K = 8. Concerning RPRM model with 5 non-empty clusters, results on cluster G are similar while posterior median of excess risk β_*F*_ and characteristics of cluster F are different. Indeed, this second cluster F not contains exactly the same number of uranium miners for different values of K. Almost 630 common miners belong the second cluster for all fixed number K except for K = 5 where there are approximately 250 miners more (cluster F in Supplementary Figures 1, 2). When comparing the 630 commons miners to these 250 miners, we notice that common miners received a higher cumulative exposure to radon and they were all working in other mines than Hérault's one. The 250 miners who differed with K = 5, have lower cumulative exposure to radon and slightly more than half of them worked in Hérault's mine. Finally, there are only two cases of lung cancer death among these 250 miners. The risk associated to the 630 common miners is also higher than that associated to 880 miners belonging the second cluster with the partition in 5 non-empty clusters. Consequently, this second cluster F is again significant or nearly significant with partitions in 6 or 7 clusters but not with the partition in 5 non-empty clusters. The posterior median of β_*F*_ is estimated near to the same value for 6 and 7 clusters than 8 clusters but near 0.3 for model with 5 clusters. Despite these differences, this second high risk cluster exists for all models with very near characteristics, in particular with less important cumulative exposures to radon, γ-rays and uranium dust exposure but with young age at the start of work.

We do not systematically observe an increasing risk corresponding to increasing exposure levels. It is particularly the case when focusing on cluster B (Figure 5). This cluster is associated to the second lowest risk whereas the miners in this cluster are highly exposed. The main differences compared to other clusters are the important proportion of uranium miners working in Hérault's mine and the period after mechanization. Modeling association between profiles and mortality allows to obtain finer interpretation of effect of exposure levels than studies including direct associations with exposures could not have done.

## 4. Discussion

In this work, we developed an original Bayesian PRM model based on an instantaneous excess hazard ratio model as disease submodel and a truncated Dirichlet process mixture as attribution submodel. This model was applied to the estimation of the lung cancer mortality associated with multiple cumulative exposures to ionizing radiations as well as any other occupational exposures through proxy variables (i.e., job types and localization of the mines). An adaptive Metropolis-Within-Gibbs algorithm, including three label switching moves, was implemented in Python to sample from the joint posterior distribution of all the unknown parameters and latent variables. Simulations were performed in order to validate the implemented algorithm (Results can be found in the Supplementary Material).

After fitting our full Bayesian PRM model to the post-55 sub-cohort of French uranium miners, the target posterior distribution was suspected to be highly multi-modal and our MCMC algorithm to converge to local modes. Consequently, Bayesian RPRM models were also fitted to the post-55 sub-cohort, where the number K of non-empty clusters was fixed to 5, 6, 7, and 8. In this paper, we focused on the results provided by the Bayesian RPRM with 8 non-empty clusters (including the cluster of non-exposed miners) that led to very interesting clusters of miners. Two of them were associated with a strictly positive and statistically significant EHR of death by lung cancer. The first group (EHR = 1.4, 95%IC = [0.60, 2.60]) corresponded to the miners the most highly exposed to radon, gamma rays and uranium dust and for more than 19 years (mainly before mechanization or as hewer after mechanization not in the mine located at Herault). The second group (EHR = 1.2, 95%IC = [0.17, 2.80]) corresponded to the miners who were very young when first exposed and who were highly exposed to radon, gamma rays and uranium dust for more than 13 years (mainly hewer after mechanization or other underground job before mechanization). Finally, the model showed that the group of miners who worked after the mechanization and mainly in the mine located at Herault (the only included uranium mine with sedimentary soil) had the second lowest risk whereas the miners in this cluster were highly exposed. Thus, this Bayesian RPRM model allowed providing an original, rich and fine interpretation of the potential association between the risk of death by lung cancer and specific radiation exposure profiles of French uranium miners, especially by modulating the effect of radiation co-exposures by other information, such as age at first exposure and duration of exposure. Results with the three other possible values of K from 5 to 7 are described in Supplementary Material.

Unfortunately, the target posterior distribution of our full Bayesian PRM model was suspected to be highly multi-modal, given the data available in the post-55 sub-cohort of French uranium miners. This could be due to a lack of signal in the database avoiding to strongly highlight, if it exists, an “optimal” partition of uranium miners (i.e., with the highest posterior probability). Additionally, the Bayesian PRM models have a large number of parameters and latent variables and, thus, in the specific context of a lack of signal in the available data, applying a MCMC algorithm might not be the most suitable Bayesian inference. As illustrated by Gelman et al. (64), due to the random walk of Gibbs sampler and Metropolis algorithm, the simulations can take a long time before moving to the target distribution. Particularly, for complex models with high dimensional target distribution, a random walk can remain local. Betancourt and Girolami (65) also illustrated that Gibbs samplers and Metropolis-Hastings algorithms explore the target distribution slowly, and it get worse when the number of groups or levels increases. Although difficult to tune, Hamiltonian Monte Carlo (HMC) (66) algorithms may be more efficient than adaptative Metropolis-Within-Gibbs algorithms to fit Bayesian PRM models (65).

Other limitations, which are specific to our case study, open new avenues for methodological research in Bayesian PRM models. First, in this paper, we only considered the sum of exposure measurements collected for each covariate, over the entire career of each miner. The Bayesian PRM models could be extended to take into account the temporal dynamics of multiple exposures. Each individual could be assigned to a unique cluster that would depend on his whole trajectory of exposure. Alternatively, the class label of each individual could change over time depending on the temporal dynamics of his exposures. Secondly, this study does not account for the tobacco consumption of miners whereas it is known to be the most important cause of lung cancer. The smoking status is only available for 4.2% of the miners in the post-55 sub-cohort of French uranium miners. This major lack of information makes it very unreliable to adjust for smoking status when estimating the risk of death by lung cancer due to multiple exposures. It makes it also very unreliable to impute about 96% of smoking status given that no potential predictors for smoking status are available in the French cohort of uranium miners. Actually, if tobacco consumption is the main responsible for the excess hazard ratio of death by lung cancer in the French cohort of uranium miners then a higher proportion of smokers should be observed in the clusters with high excess hazard ratio compared to the ones with low excess hazard ratio (and reciprocally). Given the available data, this does not appear to be the case. The ratios between the number of smokers and the number of non-smokers for clusters A, B ,C, D, E, F, G (defined in Figure 4) are 12/3, 7/0, 14/5, 17/4, 5/5, 16/8, 34/12, respectively, where clusters F and G have the highest excess hazard ratios of death by lung cancer. The associated proportions of smokers for clusters A, B ,C, D, E, F, G are 0.8, 1.0, 0.74, 0.81, 0.50, 0.67, 0.74, respectively. Of course, these estimated ratios must be interpreted with caution given the limited available data (i.e., 142 miners with smoking status data). Nevertheless, previous analyses on the impact of smoking in occupational cohort studies of uranium miners suggested that smoking was not a source of confounding in these studies (67). This is not surprising since there is actually no strong reason to think that the smoking status is strongly associated with occupational exposure levels. Interestingly, if the proportion of missing smoking status was reasonable (about 30%). The Bayesian PRM models could deal with these missing covariates while accounting for their associated uncertainty to identify exposure profiles. Note that our results should be interpreted with caution given the small number of death by lung cancer in the post-55 French cohort of uranium miners and the lack of data about the tobacco consumption of French uranium miners. As a third limitation of our study, exposure measurement error on radon, γ-rays and uranium dust was not accounted for when identifying the clusters and estimating the associated risks of death by lung cancer. However, complex structures of measurement error were identified in the French cohort of uranium miners (48, 53, 68). It is also well-known that exposure measurement error questions the validity of statistical inference in epidemiological studies (69, 70). When it is not or only poorly accounted for, it may lead to biased risk estimates, a loss in statistical power and a distortion of the exposure-response relationship. Owing to their hierarchical structure, the Bayesian PRM models could be extended to account for exposure measurement error which is, with multicollinearity, one of the most important issues when assessing exposome-health associations (21).

Defining and monitoring the human exposome is a strongly difficult task, given the wide variety of environmental factors, biological endpoints and gene-environment interactions (4, 6, 22). Wild suggested that measuring exposure in any one of the following broad exposure categories—internal (e.g., hormones, microflora), specific external (e.g., toxicants) and general external (e.g., social, psychological)—can reflect certain aspects of the overall exposome (5). Moreover, following Bennett et al. (71), it can be advantageous for the development of statistical methods to narrow the focus of the exposome to a particular class of exposures or/and specific life stages as a way to improve and validate them to apply them later to the broader exposome concepts in a risk assessment or regulatory framework. This was the case in this work that focused on occupational exposure to several types of ionizing radiations of French uranium miners, considering only a small number (i.e., 7) of exposure covariates. This paper shows that the PRM models are promising for exposome research in this context. Interestingly, they could also guide some extensions for higher dimensional data. A great number of covariates including environmental and genetic risk factors could be included in the PRM models in order to study, for instance, gene-environment interactions but the performances of the PRM models should then be assessed in this more challenging context.

## Data Availability Statement

The data analyzed in this study is subject to the following licenses/restrictions: data availability is restricted due to subject anonymity. Requests to access these datasets should be directed to klervi.leuraud@irsn.fr.

## Ethics Statement

The studies involving human participants were reviewed and approved by Comité Consultatif sur le Traitement de l'Information en matière de Recherche dans le domaine de la Santé (CCTIRS) and Commission Nationale de l'Informatique et des Libertés (CNIL). Written informed consent for participation was not required for this study in accordance with the national legislation and the institutional requirements.

## Author Contributions

SA and CG contributed the conception and design of the study. MB performed the statistical analysis under the guidance of SA and CG. MB wrote the first draft of the manuscript. MB, OL, SA, and CG contributed to the results evaluation and interpretation. MB, CG, and SA wrote the sections of the manuscript. All authors contributed to manuscript revision, read, and approved the submitted version.

## Conflict of Interest

The authors declare that the research was conducted in the absence of any commercial or financial relationships that could be construed as a potential conflict of interest.
