# Supplementary material for: Bayesian Profile Regression to Deal With Multiple Highly Correlated Exposures and a Censored Survival Outcome. First Application in Ionizing Radiation Epidemiology
Source: Front Public Health. 2020 Oct 27;8:557006. doi: 10.3389/fpubh.2020.557006 (PMC7652768; doi:10.3389/fpubh.2020.557006)
Supplement: Supplementary file 1 [file Data_Sheet_1.PDF]

Supplementary Material

1 RESULTS FOR THE BAYESIAN PROFILE REGRESSION MIXTURE RPRM MODELS ASSUMING 5, 6 AND 7 NON-EMPTY CLUSTERS

Figures S1, S3 and S5 display the number of French uranium miners (top left), the number of deaths by lung cancer (bottom left) and the instantaneous excess hazard ratio (per 100 WLM) of death by lung cancer (β) in each cluster, when fitting a Bayesian RPRM model assuming 5, 6 and 7 non-empty clusters respectively. The boxes represent the three quartiles (1st quartile, median and 3rd quartile) of the approximate posterior distribution of β and the whiskers of the boxplots show the 95% posterior credible interval for β for each cluster. The black horizontal line is 0 displaying an absence of instantaneous excess risk. The red boxplots display the clusters with a significant increased excess risk of death by lung cancer (i.e., the associated 95% credible interval is higher than 0), and the blue boxplots display the clusters without a significant excess risk (i.e., the associated 95% credible interval contains 0).

Figures S2, S4 and S6 allow characterizing the exposure profiles associated to each cluster, when fitting a Bayesian RPRM model assuming 5, 6 and 7 non-empty clusters respectively. Each column is associated to one exposure variable of interest, when estimating the risk of radiation-related lung cancer in the French cohort of uranium miners. From left to right: γ-rays exposure (in milliSieverts), radon exposure (in Working Level Months), uranium dust exposure (in Becquerel per hour per cubic meter), job type, type of mine (i.e., sedimentary mine located in Herault vs other granitic mines located in metropolitan France), age at first exposure (in years) and duration of exposure (in years). The job types are the following: 1) hewers before mechanization, 2) hewers after mechanization, 3) other underground work before mechanization, 4) other underground work after mechanization and 5) surface work. The boxes represent the three quartiles (1st quartile, median and 3rd quartile) of the approximate posterior distribution of the parameters defining the probability distribution followed by each variable in each cluster and the whiskers of the boxplots represent the associated 95% posterior credible intervals. The black horizontal line on each sub-graph of a given parameter θ represents $\tilde{\theta}$ which is the median of the posterior medians of all clusters. The colors of the boxplots help to compare the values of parameters in the different clusters. In fact, when the 95% credible interval of a given parameter in a given cluster contains the value $\tilde{\theta}$, the box is blue. When the credible interval is higher than $\tilde{\theta}$ then the boxplot is red, and if it is lower than $\tilde{\theta}$ then the boxplot is green. The clusters are ordered in ascending order of the approximate posterior median of each risk coefficient $\beta_c, c \in \{1, C_{max}\}$ of death by lung cancer in the French cohort of uranium miners.

2 SIMULATION STUDY

The purpose of this simulation study was to validate the performance of the implemented MCMC algorithm to fit our Bayesian RPRM model, from 100 simulated data sets.

Each data set is simulated according to a Bayesian RPRM model assuming 4 non-empty clusters (among which a cluster of non-exposed individuals) from a population of 200 uranium miners. For each dataset, 4 groups of miners were generated from a population of 200 miners. Like in the post-55 sub-cohort of French uranium miners, almost 5% of uranium miners were assigned to the cluster of unexposed individuals. The exposed miners were then divided into 3 clusters according to the following proportions: 1/6, 1/3 and 1/2. The "true" values of the parameters (see Figure 2) considered to simulate the data can be found in Table S1 (except for α as we considered the restricted version of the PRM model in which the number of clusters is fixed). We then conducted the Bayesian inference of the same RPRM model (i.e., the model

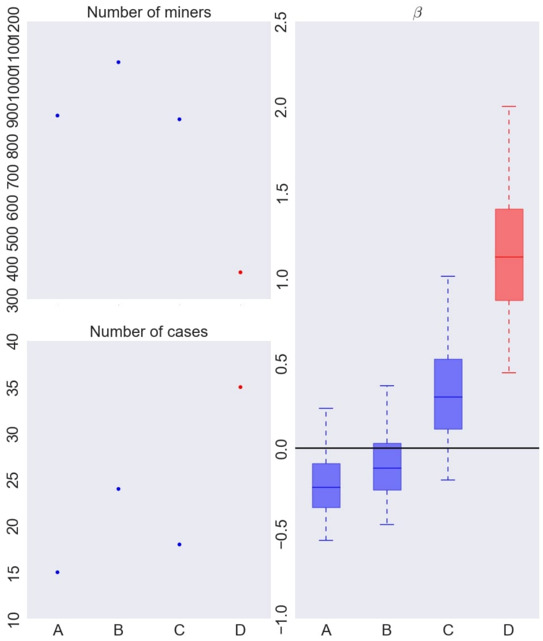

Figure S1. Number of French uranium miners (top left), number of deaths by lung cancer (bottom left) and instantaneous excess hazard ratio (per 100 WLM) of death by lung cancer (β) in each cluster (right), when fitting a Bayesian RPRM model assuming 5 non-empty clusters from the French cohort of uranium miners. The cluster including non-exposed miners is not displayed.

used for data generation was the same as the fitted model) from each of these 100 datasets using our adaptive Metropolis-Within-Gibbs algorithm. Our aim was only to validate our MCMC algorithm. The 95% coverage rates are defined as the percentage of 95% posterior credible intervals which contained the ”true” value of each parameter (see table S1). These coverage rates should be as close as possible to the nominal rate of 95%. This seems correct in our simulations.

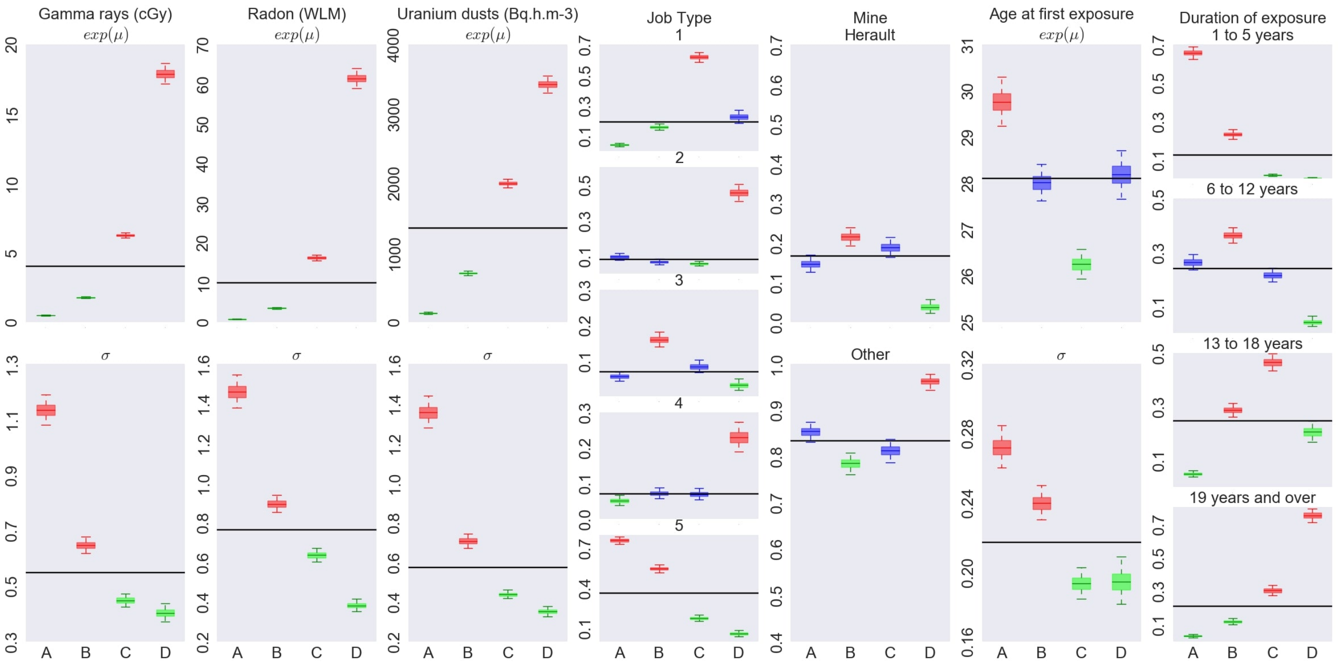

Figure S2. Characterization of the exposure profiles associated to each cluster, when fitting a Bayesian RPRM model assuming 5 non-empty clusters.The cluster including non-exposed miners is not displayed.

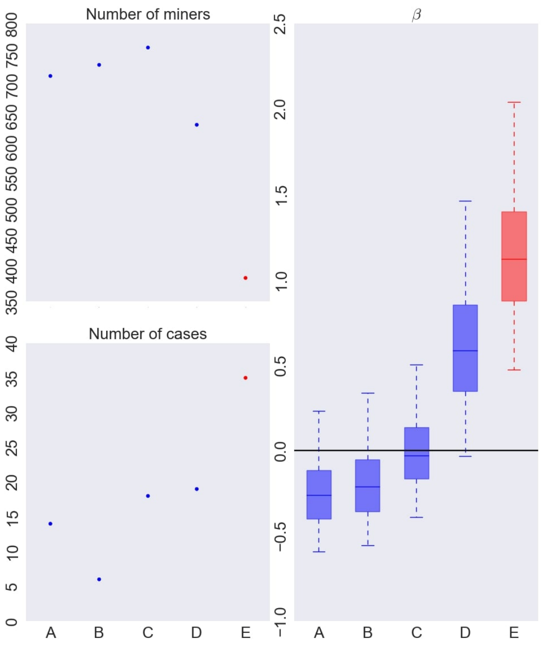

Figure S3. Number of French uranium miners (top left), number of deaths by lung cancer (bottom left) and instantaneous excess hazard ratio (per 100 WLM) of death by lung cancer (β) in each cluster (right), when fitting a Bayesian RPRM model assuming 6 non-empty clusters from the French cohort of uranium miners. The cluster including non-exposed miners is not displayed.

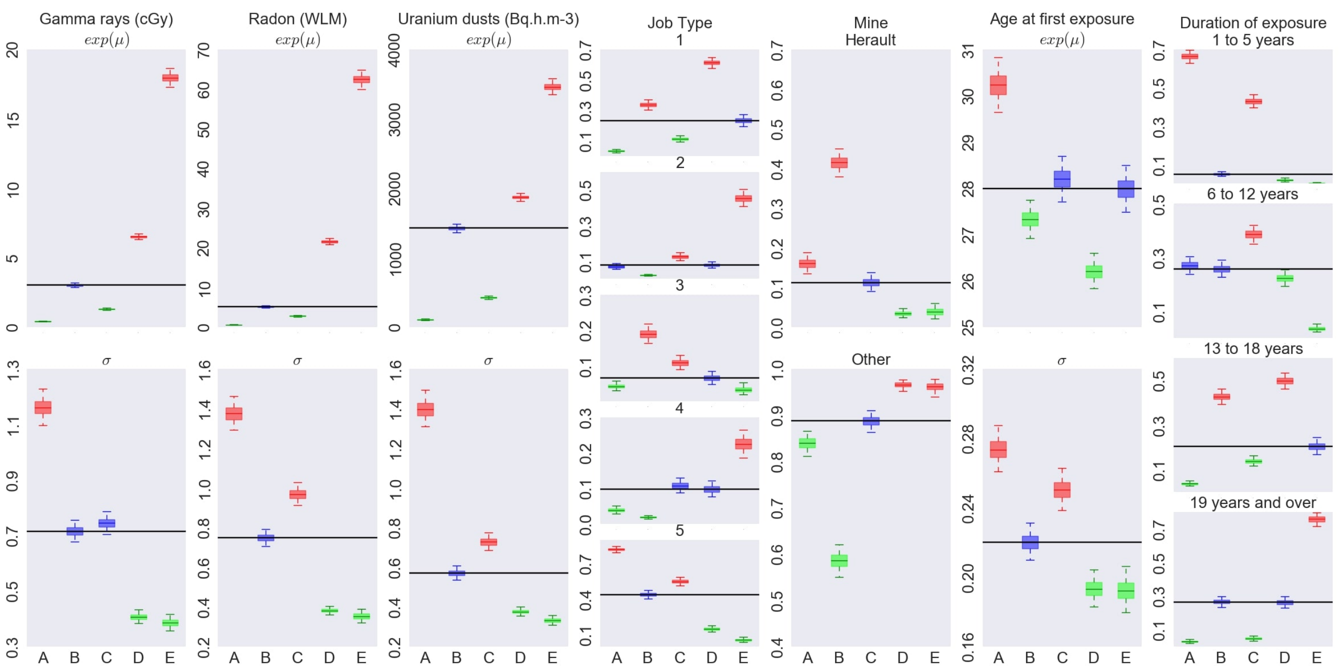

Figure S4. Characterization of the exposure profiles associated to each cluster, when fitting a Bayesian RPRM model assuming 6 non-empty clusters.The cluster including non-exposed miners is not displayed.

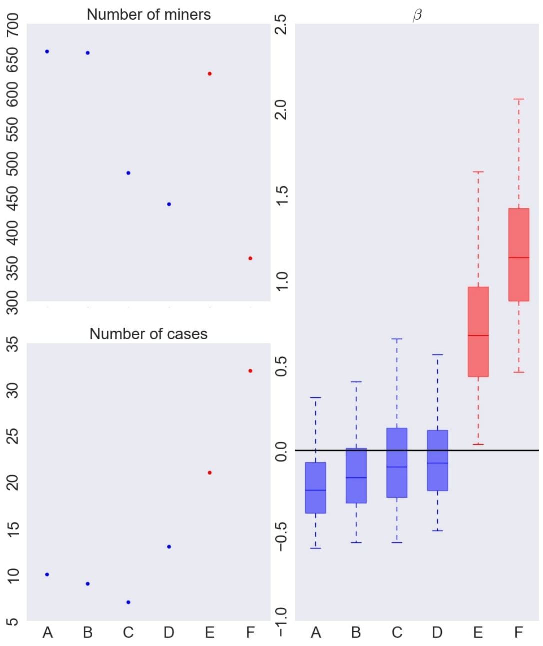

Figure S5. Number of French uranium miners (top left), number of deaths by lung cancer (bottom left) and instantaneous excess hazard ratio (per 100 WLM) of death by lung cancer (β) in each cluster (right), when fitting a Bayesian RPRM model assuming 7 non-empty clusters from the French cohort of uranium miners. The cluster including non-exposed miners is not displayed.

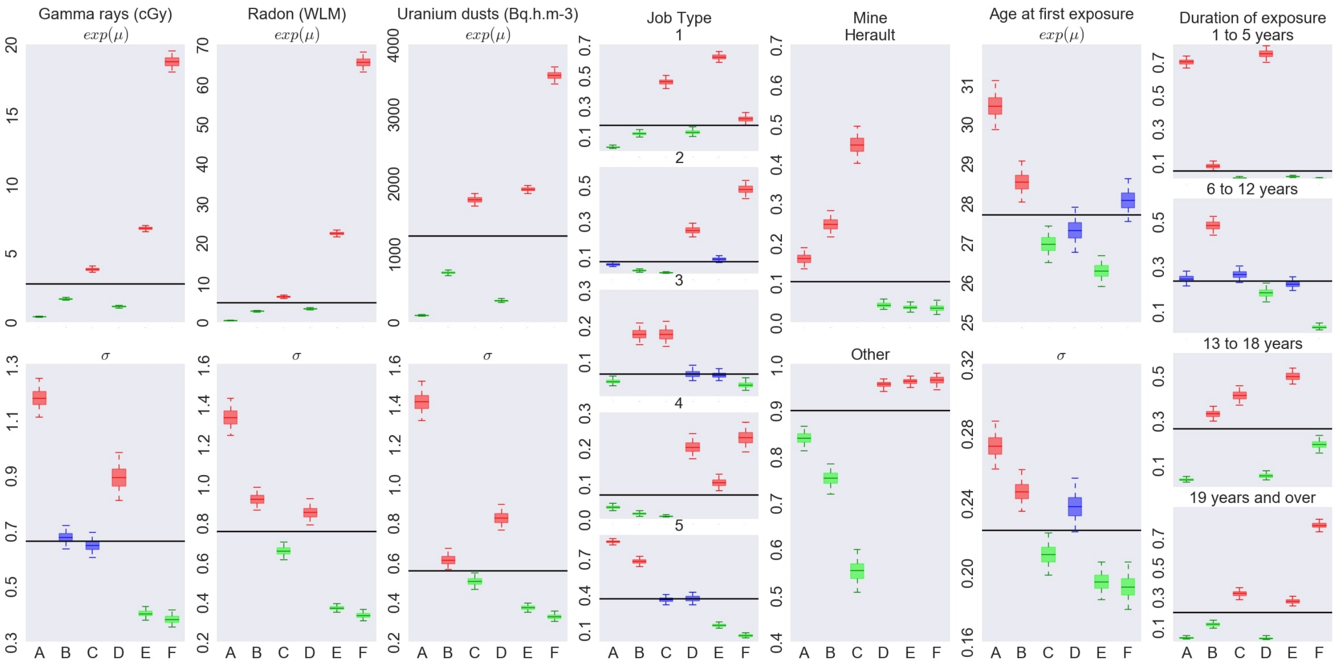

Figure S6. Characterization of the exposure profiles associated to each cluster, when fitting a Bayesian RPRM model assuming 7 non-empty clusters.The cluster including non-exposed miners is not displayed.

Parameters	"True" value			95% coverage rate		
	Cluster 1	Cluster 2	Cluster 3	Cluster 1	Cluster 2	Cluster 3
β	10	30	50	96	96	86
μ^A	2	6.5	11	95	95	93
μ^G	-4	0	4	97	93	97
μ^P	2	6.5	11	93	96	94
μ^R	-2	3	8	96	95	93
$p^{J,0}$	0.10	0.15	0.20	93	90	90
$p^{J,1}$	0.20	0.25	0.30	98	92	94
$p^{J,4}$	0.35	0.225	0.10	91	90	95
$p^{J,2}$	0.30	0.175	0.05	90	95	95
$p^{J,3}$	0.05	0.20	0.35	97	94	94
$p^{M,NH}$	0.10	0.50	0.90	92	96	97
$p^{M,H}$	0.90	0.50	0.10	92	96	97
$p^{T,1}$	0.40	0.25	0.10	95	95	93
$p^{T,2}$	0.10	0.15	0.20	96	92	94
$p^{T,3}$	0.20	0.25	0.30	98	94	92
$p^{T,4}$	0.30	0.35	0.40	95	95	93
σ^A	0.50	0.50	0.50	96	87	96
σ^G	0.50	0.50	0.50	93	92	96
σ^P	0.50	0.50	0.50	94	91	98
σ^R	0.50	0.50	0.50	92	91	94

Table S1. True values of parameters and 95% coverage rates for 100 datasets generated according to Bayesian RPRM model assuming 4 non-empty clusters (including a cluster of non-exposed individuals) from a population of 200 uranium miners.
